# Research capacity-building of midwives expanding access to safe abortion in the Democratic Republic of Congo: transnational research collaborations with civil society organizations

**DOI:** 10.1080/16549716.2025.2545627

**Published:** 2025-08-21

**Authors:** Kirsty M Bourret, Marie Chantal Kankolongo, Nadia Lobo, Jean-Claude Mulunda, Eva Åkerman, Elisa M Maffioli, Marie Klingberg-Allvin

**Affiliations:** a Department of Women and Children’s Health, Karolinska Institutet, Stockholm, Sweden; b La Société Congolaise de la Pratique Sage-femme, Kinshasa General Hospital, Kinshasa, Democratic Republic of Congo; c Evaluation and Research, IPAS-DRC, Kinshasa, Democratic Republic of Congo; d Department of Health Management and Policy, University of Michigan School of Public Health, Ann Arbor, USA

**Keywords:** Sub-Saharan Africa, global health, sexual and reproductive health, midwives’ associations, abortion, gender equity

## Abstract

Enabling civil society organizations (CSOs) in sub-Saharan Africa to develop research ensures equitable scholarly representation and addresses local problems in sexual and reproductive health. To this end, a transnational research collaboration was formed to evaluate abortion services and develop the research capacity of two CSOs in the Democratic Republic of Congo (DRC). The team included Karolinska Institutet, Partners for Reproductive Justice (IPAS) and the National Midwives’ Association (SCOSAF). The objective of this article is to discuss the context of the partnership, including research capacity-building inputs, processes, outputs, and outcomes, as well as lessons learned and recommendations. Activities were tailored to the research capacities of each CSO and the research team of clinical care providers over the two-year project period. Research capacity-building resulted in increased opportunities for team members to conduct research and manage research projects outside of academia. Overall, there were improvements in midwives’ capacity to design and conduct research, and in the midwives’ association’s capacity for research management and project administration. Recommendations for others include pragmatic incorporation of gender considerations, approaches to organizational and individual research capacity-building, and baseline CSO capacity assessments for research management. Health research conducted in a non-academic setting, specifically within CSOs, can be a pathway to research equity. In DRC, it strengthened health professionals in their capacity to generate evidence to influence local abortion policy and health services in Kinshasa, DRC.

## Paper Context

**Main findings:** Approaches to research capacity-building outside academia and within midwives’ associations contribute to gender equitable research development and generation for and by African-led clinician researchers, including mostly women-led professions such as midwives.

**Added knowledge:** Promoting gender transformative research collaborations begins with considerations of female leadership and female professional development in research capacity-building such as fostering safe learning spaces and minimizing economic impacts by ensuring remuneration and pay equity.

**Global health impact for policy and action:** Embedding research capacity-building within projects involving midwives and service delivery, especially when coordinated through midwives’ associations, can serve as an effective gender-equitable strategy to enhance locally driven evidence. This approach supports the sustainable integration of midwifery competencies and advances improvements in health service delivery, including person-centred comprehensive abortion care.

## Background

Achieving the United Nations’ Sustainable Development Goals (SDGs) and improving sexual and reproductive health and rights (SRHR) requires high-quality research to guide policy, implementation, and practices. Global health research is now being understood as an ‘outcome of globally shared risks and responsibilities that require collective action to achieve good health for all’ [1]. Historically, approaches to global health research were rarely rooted in equity. These legacies, colonial in nature, continue to perpetuate power imbalances in global health research by shaping ‘who’ influences and dominates the field [2].

Presently, there is a significant underrepresentation of researchers based in sub-Saharan Africa in global health research outputs, particularly in health-related fields [3]. Barriers such as inequitable access to research funding, grant eligibility, and a lack of mentorship lead to a dependency on foreign organizations, consequently eroding locally led research advancements and development. Furthermore, these issues inequitably impact Africa-led health researchers based on gender and discipline [4]. Between 2014–2016, women^1^^1^We use the term woman/women as most of the research and data in our manuscript is related to pregnant or non-pregnant cisgender women [14]. represented less than one-third of all first authors in sub-Saharan Africa [4]. While poor knowledge production impacts African-based researchers’ capacity to address health challenges, not asking ‘who’ generates this knowledge is also problematic when advancing SRHR [3].

Transnational collaboration with embedded research capacity-building is an optimal approach to improve and sustain knowledge production that centers Africa-led research development [5]. Fostering opportunities outside academia to include communities and the civil society sector is an additional strategy to ensure research is equitable, including gender representation [3]. Typically, capacity-building for research management and support in Sub-Saharan Africa is understood and applied within academic or research institutions rather than within the civil society sector [6,7]. Civil Society Organizations^2^^2^We use the definition of CSO put forward by the United Nations Research Institute for Social Development, which is a broad understanding of the term to capture an organization outside of the state and operating as a non-profit, including non-governmental organizations and the full range of associations (e.g. professional associations, trade unions, cultural and religious groups) [8]. (CSOs) have a different organizational structure and purpose than academic institutions. Yet, given their networks and relationships to a given population, CSOs can effectively address health inequities and are well-positioned to be involved in research at various stages, such as priority-setting, operationalization or knowledge exchanges [9–11]. Additionally, research capacity-building can be transferred to CSOs’ overall capacity in monitoring and evaluation, generating evidence for stakeholders, and resource mobilization [12,13].

Midwives’ associations are an example of mainly women-led CSOs [13]. Research conducted by midwives’ associations strengthens the profession’s ability to advocate and influence the advancement of the profession and impact SRHR [12,13]. Midwives and nurses make up almost half of the global health workforce and provide almost ninety percent (90%) of primary rural health services in many sub-Saharan countries [15]. Research contributions by these professions, particularly in resource-constrained settings, impact the delivery of quality healthcare across health systems [15,16]. Yet, as women-dominated professions, their research contributions are disproportionately limited compared to male-led professions [17]. For example, in 2023, fewer than ten percent (10%) of authors with six or more publications in scientific journals in SRHR were nurses or midwives [3].

In Sub-Saharan contexts, academic-based research capacity-building can be valuable to support midwifery educators and researchers, yet due to gender biases, many midwives struggle for opportunities to enter the academic space, thus remaining on the periphery [15–20]. There is therefore a strong rationale to support all and any midwife who expresses an interest in research development [17–19]. Collaborative approaches with non-academic midwife entities, such as midwives’ associations, can be an additional strategy for increasing the number of midwife researchers [13]. Yet, given the limited resources midwives’ associations often face in many contexts, collaborations must equally consider how to strengthen the organizational capacity of the association to support their midwife researchers and thus ensure meaningful and sustained contributions in health research, policy and practice [13–20].

Given the importance of supporting and encouraging others in the equitable development of Africa-led research, the objective of this paper is to share the story of a transnational collaboration and research capacity-building of two CSOs in DRC namely Partners for Reproductive Justice Democratic Republic of Congo (IPAS-DRC) and the National Midwives’ Association (Société Congolaise de la Pratique Sage-Femme: SCOSAF). We begin by providing the context of the research capacity-building, including a summary of abortion access in DRC and the aligned research objectives. We then discuss the origins of the transnational collaboration, the CSOs’ base organizational capacities, and how gender influenced the research capacity-building approach. Research capacity-building inputs, processes, outputs and outcomes are explored as well as lessons learned. Finally, we provide recommendations for other transnational collaborations, particularly with midwives’ associations.

## DRC and access to abortion

Deaths attributable to unsafe abortion account for 5.1% to 17.2% of all deaths on the African continent [21]. In DRC, access to abortion has long-standing legal restrictions, and unsafe abortion practice is considered high [21]. Until 2018, pregnancy termination in DRC was legally restricted under the Belgian Penal Code, punishable by up to 15 years of incarceration [22]. The African Charter on Human and Peoples’ Rights on the Rights of Women in Africa, also known as the Maputo Protocol, is a legal instrument for African countries to improve access to abortion and post-abortion care [23]. In 2018, DRC complied with the Maputo Protocol, permitting abortion if the woman is a victim of rape or assault or if the pregnancy endangers her mental or physical health, her life or that of the foetus. Following the law, the Ministry of Health created guidelines for providers to implement evidence-based abortion care [24,25].

IPAS-DRC is the national office for IPAS International, a non-governmental organisation whose mission is to use a comprehensive approach across sectors to build abortion ecosystems. Since 2019, IPAS-DRC and the Ministry of Health have worked together to implement the new abortion legislation by strengthening the capacity of the health system to offer person-centred comprehensive abortion care [25]. CAC comprises information about abortion, abortion management, contraceptive counselling, and post-abortion care for incomplete abortion [26]. Person-centred CAC is defined as CAC that is respectful and responsive to the needs and values of the client [27]. At the time of the research, IPAS-DRC supported 34 health facilities in DRC’s capital city, Kinshasa, the location of the project. Health facilities provided basic or comprehensive abortion care.^3^^3^The WHO has historically recommended the use of signal functions to assess the quality of CAC in health facilities. These are specific structural and process indicators delineated based on the types of CAC services a health facility can provide. There are two categories: basic and comprehensive. Basic CAC services are comprised of the following six signal functions: perform induced abortion up to 12 weeks’ gestation, provide post-abortion contraception, administer essential antibiotics, administer intravenous fluids, administer oxytocics and remove retained products of conception for uterine sizes up to 12 weeks. Comprehensive CAC includes the six signal functions of basic care plus an additional four: perform induced abortion for uterine sizes greater than 12 weeks, provide PAC for uterine sizes greater than 12 weeks, perform blood transfusion and perform laparotomy [28]. IPAS-DRC provided support for training, access to IPAS-DRC mentors, equipment, and supplies [24]. According to IPAS-DRC, facility registers documented 2,860 abortions across the 34 facilities from January to June 2022. Congruent with the 2022 World Health Organization (WHO) abortion care guideline, IPAS-DRC and the Ministry of Health had incorporated a diversity of providers, including midwives, in their standards and training [29,30].

IPAS-DRC worked closely with local stakeholders, including local CSOs and academia, to collect and implement context-specific evidence to ensure sustainable programming and scale up access to safe abortion in the country. The National Midwives’ Association, SCOSAF, had been an active stakeholder in IPAS-DRC’s implementation activities of the Maputo Protocol and had ensured that the national guidelines on abortion care, as well as the national midwifery education curriculum, had incorporated considerations for midwife-led CAC. Furthermore, SCOSAF’s leadership had received training as CAC educators and mentors, providing ongoing continuing education and support for their members.

## Research objectives

**Figure 1.**
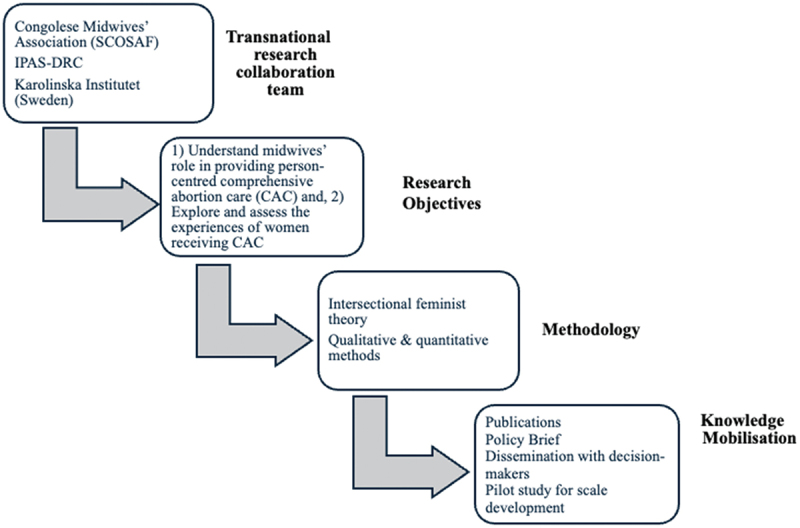
Description of partnership and research process.

The purpose of the research was to measure and understand the impact of IPAS-DRC’s implementation activities as they related to midwives and women accessing CAC under the new legislation. From the aim, the transnational research team created two research objectives: 1) To understand midwives’ role in providing CAC in the DRC health system, and 2) To explore and assess^4^^4^This research objective was then divided into a qualitative and quantitative study. The quantitative study was piloted for scale development only and was designed to 1) measure women’s experiences and costs of CAC, 2) measure the costs of abortion to health facilities. women’s experiences receiving CAC in Kinshasa, DRC. See Figure 1 for a description of the partnership and research process.

Pertaining to the first research objective, integrating midwives as autonomous abortion providers is recognized as an essential strategy for reducing critical health system gaps, increasing the availability of abortion and contraceptive methods [31–33]. Before the change in abortion legislation, midwives in DRC had been educated and trained in the management of incomplete abortions in emergencies. A previous collaborative study conducted by the principal investigator (KB) and SCOSAF demonstrated that while midwives wanted to implement abortion care, system barriers resulted in only one third of trained midwives integrating this skill [34]. As midwifery training now includes all components of abortion care there was a need to re-evaluate effective and sustainable approaches to integrate midwife-led abortion care in DRC.

The second research objective related to women’s experiences of CAC since the legal ratification. Before ratification, studies in DRC demonstrated that women who had clandestine abortions would avoid health facilities for fear of stigma and mistreatment [21,35]. A key rationale for person-centred CAC is that it fosters an enabling environment for abortion care. This could lead to an increase in the use of health services, decreasing the number of women seeking care for unsafe abortions outside of facilities [21,36]. Given the government’s commitment to the integration of CAC, it was seen as a priority to evaluate the experiences of women receiving abortion care from centres that had received health service support in the implementation of CAC [24].

## Transnational research collaboration

In 2021, IPAS-DRC, SCOSAF, and a group of researchers based at Karolinska Institutet (KI) formed a transnational research collaboration. Historically, individuals from each of the institutions have worked together in various types of projects and collaborations. This was the first time the group formed an official partnership. The roles and responsibilities of all three institutions were: IPAS-DRC, as the primary implementation partner; SCOSAF, as the sub-implementation partner responsible for research management and support; and KI, providing oversight and support to the research and research capacity-building.

Within the partnership, individuals from each CSO formed an operational research team based in DRC. This included a member of the research, monitoring and evaluation team at IPAS-DRC (NL), nine health providers affiliated with IPAS-DRC as data collectors, and midwife members of SCOSAF including the coordinator of research operations (MCK) and three research assistants. The principal investigator (KB) was the coordinator of the research and research capacity-building with SCOSAF as part of her post-doctoral fellowship at KI. The operational research team in DRC worked in close consultation with KI and the direction of IPAS-DRC (EMM, EA, MCK, JCM).

## CSOs’ base capacity

In terms of base CSO capacity, SCOSAF’s organizational capacity, like many midwives’ associations globally, faced challenges related to financial and human resources stability [13,20]. Although their membership spanned all twenty-six (26) provinces of DRC, their day-to-day operations relied on volunteers and one income stream (midwife membership fees) [13]. Finally, most of their executive leaders and staff worked as midwives in the health system in various other roles (i.e. educators, clinicians, etc) and were only able to dedicate part of their time to the organization’s operations. Before and during our collaboration, SCOSAF had received project funding from the Government of Canada and the Canadian Association of Midwives and subsequently benefited from organizational capacity-building activities and program support. Therefore, at the time of this research project, SCOSAF was experiencing a diversification of income streams and financial stability for staff. As SCOSAF was conducting research within the organization for the first time, they were open to incorporating research capacity-building objectives. In contrast, IPAS-DRC, was a better-resourced CSO and a branch of an NGO based out of the USA, i.e. IPAS-International. At the time of the project, IPAS-DRC had approximately twenty-five (25) employees, including a team dedicated to monitoring and evaluation of their projects.

### Research capacity-building: inputs, processes, outputs and outcomes

Research capacity-building activities were flexible and accommodating of many individuals with varying levels of research experience. The evaluation of the project was therefore process-based and iterative. The four levels of assessment: inputs, processes, outputs, and outcomes results are presented in Table 1using a systems program evaluation framework (Table 1) [37,38]. These four levels are divided into three phases of the research: planning, operationalization, and knowledge exchange. Cross-cutting all levels are gender considerations to promote gender equity within the midwives’ association and midwife-led research [11].

**Table 1.** Evaluation framework of the research project based on Hyder et al. [37].

Research Phases Levels	Planning	Operationalization	Knowledge exchange
Inputs	Meetings with collaborators MOUs Partnership agreement Networking to build interest and support	Mentor-mentee researchers Training materials Research infrastructure including budget to pay salaries of midwife researchers and provide supplies to SCOSAF (computers, software, transportation fees, etc.)	Funding for SCOSAF to disseminate results with membership Funding from IPAS to facilitate exchange of policy brief and results to decision-makers Trained professionals Mentor-mentee researchers
Process	Research and budget planning Selection of DRC research team with gender equity lens Teaching/training regarding planning and coordination of research projects	In person and virtual teaching/training for research coordination, data collection tools, data collection, data analysis In person and virtual mentorship Applied research experience, research coordination, collection, and analysis	Co-authorship on reports for funders, policy brief, and manuscripts Networking with funders and health system actors about the results and presenting policy brief to stakeholders Website- social media highlighting research
Outputs	Four midwives (2 women, 2 men) trained and mentored in research proposal and REB development and writing SCOSAF executive mentored and knowledge acquisition in research management protocols and guidelines Two members of IPAS mentored on supporting SCOSAF on research implementation Funds received for research operations and salaries for midwife researchers	Four midwives (3 women) and 9 other care providers (5 women) trained, mentored and experience with development of data collection tools, testing tools, data collection and analysis. Data management IPAS-DRC experience with supporting SCOSAF in data collection and analysis SCOSAF staff mentored and knowledge acquisition on fiscal management of research and reporting	Two midwives (1 woman) co-authoring 3 publications 2 IPAS-DRC team co-authoring 3 publications Research and Evaluation team IPAS-DRC and SCOSAF co-writing policy brief Four midwives (3 women), SCOSAF executive and IPAS-DRC co development of stakeholder exchanges and knowledge sharing to leverage research results in the health system SCOSAF staff co-producing final reports to IPAS-DRC and funders
Outcomes	Midwives and CSO’s capacity to secure research collaborations, including funding acquisition and research planning Ability of the CSOs to incorporate gender equity approaches to partnerships and research	Two evidenced-based locally driven research studies regarding CAC, person-centred care and midwifery	Career development of two midwives (1 woman) in their post-graduate studies Enhanced capacity of SCOSAF and IPAS-DRC to manage research projects and apply their research evidence to influence policy and practice with midwives and decision-makers regarding CAC in DRC

#### Inputs and processes

A systematic review of non-academic research capacity strengthening training models in sub-Saharan Africa showed that interventions are more likely to succeed when they take place over extended periods and involve mentorship [9]. Therefore, our approach was tailored to ensure adequate time and equitable processes to meet the team’s needs and levels of knowledge. In-person training took place at various points during the project, and virtual training and ongoing mentorship were undertaken when the principal researcher (KB) could not be on-site. Training was pragmatic, flexible and involved smaller-dosed didactic sessions with one-on-one mentorship when applying theoretical content. For example, training sessions on research protocols and ethics applications were followed by jointly writing protocols and other documents for the project. Overall, this type of realist approach was also appropriate for mitigating known barriers to midwives’ participation in research, such as competing personal or professional responsibilities [9–36,38,39].

The IPAS-DRC research team was provided with mentorship and the opportunity to have hands-on experience with research ethics approval, development and piloting of data collection tools, analysis, and document writing. Like with SCOSAF, flexible support suited the IPAS-DRC staff’s other work responsibilities.

In terms of other research capacity-building inputs, infrastructural challenges common in non-academic research capacity training in Sub-Saharan Africa were addressed by ensuring the research budget included administrative support for the SCOSAF office [9]. Therefore, adequate physical space and other supports, including researcher transport, internet connection, office supplies, computers, and access to research software, were provided. Furthermore, processes were included to promote gender equity, such as the development of female leadership and professional development [11,40,41]. This included hiring women to lead the operational research team in DRC, equitable remuneration compared to men and physicians on the project, and flexible schedules to accommodate family and community responsibilities [11,40,41].

#### Outputs and outcomes

Outputs during the planning and operationalization phases were observed in terms of improved and applied research competencies at the individual and organizational levels [39]. For example, in the operationalization phase, seven midwives (six women) and six nurses and doctors (three women) received training, mentorship and practical experience with the development of data collection tools, recruitment, ethics, data collection, analysis and management. During this phase, the SCOSAF research team was mentored on the fiscal management of research and reporting.

In the knowledge exchange phase, two midwives (one woman) and two IPAS-DRC team members (one woman) co-authored three publications and co-developed stakeholder exchanges, including a policy brief. Additionally, SCOSAF staff co-produced financial and narrative reports to IPAS-DRC and funders (see Table 1).

Knowledge exchange outputs included the completion of two research studies and one pilot study.^5^^5^The qualitative research was used to develop a cross-sectional study protocol to measure experiences and the costs of CAC. This study was piloted, providing the framework for a future larger-scale study in DRC. The first publication is entitled “Stakeholder perceptions of midwife-led woman-centred comprehensive abortion care in the province of Kinshasa, Democratic Republic of Congo: a qualitative descriptive study and the second ‘Experiences of Person-Centred Comprehensive Abortion Care: A Qualitative Study Among Women in Kinshasa, Democratic Republic of Congo’. Both manuscripts are published in open-access journals and available in French and English, and, therefore, are accessible to IPAS-DRC, SCOSAF and care providers in their future work to integrate CAC in DRC [42,43].

Finally, the outcomes in each research phase contributed toward an improvement in midwives’ capacity to design and conduct research, and in the midwives’ association’s capacity in research management and administration. Two midwives are pursuing post-graduate training, while another is continuing to seek opportunities for knowledge exchanges outside academia at international and local forums. Both CSOs also improved their capacity to apply their research evidence to influence policy and practice for CAC in DRC. Gender considerations allowed for the mitigation of gender bias in research that had impacts on the careers and mental health of women [4,11,40,41].

## Lessons learned and recommendations

Transnational research collaborations between CSOs and academic institutions can foster equitable research capacity building and generate more types of African-led research [39,44]. Housing research projects within a midwives’ association is one feasible approach, particularly for generating evidence to inform policy, health services delivery and SRHR [11,12]. However, partners must be cognisant that the capacity of midwives’ associations in research collaborations is influenced by the context of midwifery, which is shaped by external forces (e.g. climate change, economic crisis) and local political, health and education system arrangements [13]. We support Bourret et al.’ recommendations for the implementation of interventions with midwives’ associations that include 1) positioning midwives within their specific political and health system’s context, 2) tailoring interventions that are midwife-led and that foster leadership skills and gender equity, 3) understanding the specific challenges and solutions for midwives’ associations to support integration of the intervention, and 4) incorporating the full range of midwifery voices throughout the process^6^^6^Midwives who were not affiliated with SCOSAF, the dominant organization in DRC, were not represented in our operational research team; thus, this is one limitation of our approach. [11].

The main successes of the project are the midwife-led, locally driven, scholarly contributions to inform policy and practice regarding abortion care in DRC and the reinforcement of research capacity for two CSOs integral to advancing SRHR. The pragmatic approach and embedding of research capacity building were particularly cited as effective and beneficial. This feedback aligns with other midwives’ associations in Africa, who emphasize the need for and importance of embedded research capacity-building into overall organizational operations [11–13]. This highly practical learning environment, with remuneration for travel, learning and work, created gender equitable and safe workspaces for those who might not otherwise have had access to research experience [19,40,41]. Unlike time challenges cited by other capacity-building interventions, the two-year-long project parameter provided multiple opportunities for ongoing training, mentorship and support, with ample time to apply newly acquired skills [9].

Project length requires adequate funding and support from funders to embed research capacity and research into CSO projects [44]. Donor and funding priorities influence how or if CSOs can implement research-based projects [9]. In Malawi, it was found that CSOs prioritized service delivery projects over research-based projects, mainly because results were generated more quickly and more likely to secure future funding with donors [45]. In our case, KI and both CSOs were eager for the opportunity to incorporate research into the work of CSOs and understood the inherent value and potential impacts. They were then able to articulate this priority to the funder, who recognized the value of incorporating research in their funding to IPAS-DRC. With the funder’s support, the team’s initial interest in incorporating research into their activities could be feasibly implemented. We recommend, if possible, that academic institutions engage in early conversations with CSOs to strategize resource mobilization with like-minded funders regarding CSO-generated research along with research capacity-building.

Project challenges that were encountered pertained to SCOSAF’s human resources capacity in the face of extenuating factors [44]. During the project, IPAS-DRC’s financial staff provided ongoing support for their colleagues at SCOSAF, while KI provided ongoing support for research operations. The benefit of this approach was such that the better-resourced local CSO, IPAS-DRC, was able to impart its financial policies and procedures. However, SCOSAF was managing parallel projects with competing financial accountabilities to multiple funders. Furthermore, an abrupt change in staff and a board election revealed that the administrative capacity was dependent on individuals no longer within SCOSAF, thus creating delays in research operations and the project.

From this experience, we recommend that midwives’ associations and research collaborators co-conduct baseline assessments to understand the CSO’s capacity to incorporate research management procedures within their core administrative and financial infrastructure [12,13]. Research capacity-building activities can then appropriately incorporate administrative and financial management while including multiple individuals to ensure institutional memory. Conducting a baseline assessment of SCOSAF’s ability to incorporate research management procedures into existing operations might have better illuminated gaps, supported a rationale to the funder for organizational-level capacity-building activities and better prepared its infrastructure for staff changeover and competing financial procedures. We would like to note, that after our project ended, the Canadian Association of Midwives was supporting SCOSAF to build their core capacity to mitigate these problems.

Most importantly, we recommend that CSO research collaborations and research capacity-building interventions consider the impacts of gender and other intersecting forms of discrimination throughout the process, to promote female leadership and professional development [11,41]. The social construction of gender impacts the value placed on women in a country, and in turn the value placed on predominately women-dominated professions such as midwifery and midwives’ associations within that society [13]. Gender norms and biases in research further impact the capacity of the profession to contribute meaningfully to local evidence and subsequent policies [4,13,40]. Supporting women’s involvement and career growth, and fostering safe spaces for all team members, such as designing the intervention to minimize economic impacts to participants (i.e. childcare, remuneration for work, and pay equity), can begin to mitigate gender inequities and power imbalances, promoting gender transformative research collaborations [12,13].

## Conclusion

Health research conducted in non-academic settings, specifically within CSOs, is crucial as it promotes global health research equity and particularly the development of Africa-led research. Midwives’ associations are local experts with an understanding of emerging research questions and the capacity to support research dissemination with their stakeholders. We discussed here a transnational research collaboration and research capacity building with IPAS-DRC and SCOSAF in the development of research evaluating the impact of the legalization of abortion in DRC. Lessons learned in this context for conducting research alongside midwives’ associations can be applied to other contexts. Foremost, embracing gender-inclusive approaches that promote time and pay equity is essential to creating stable research environments in which midwives can develop and apply research competencies. Such environments also ensure that midwives’ associations have the necessary resources to generate locally relevant, evidence-informed policies and practices. Funders are encouraged to support transnational research collaborations, both within and beyond academic institutions, as a key strategy for fostering sustainable, Africa-led research ecosystems. Midwives’ associations are at the forefront of advancing gender-transformative and anti-oppressive approaches in global health research, and transnational partnerships play a critical role in enabling and amplifying these locally driven efforts.
